# Clinical implications of inflammation in atheroma formation and novel therapies in cardiovascular diseases

**DOI:** 10.3389/fcell.2023.1148768

**Published:** 2023-03-16

**Authors:** Shivan Barungi, Pablo Hernández-Camarero, Gerardo Moreno-Terribas, Rafael Villalba-Montoro, Juan Antonio Marchal, Elena López-Ruiz, Macarena Perán

**Affiliations:** ^1^ Department of Health Sciences, University of Jaén, Jaén, Spain; ^2^ San Cecilio University Hospital of Granada, Granada, Spain; ^3^ Tissue Establishment, Centro de Transfusión de Tejidos y Células, Córdoba, Spain; ^4^ Centre for Biomedical Research (CIBM), Biopathology and Regenerative Medicine Institute (IBIMER), University of Granada, Granada, Spain; ^5^ Instituto de Investigación Biosanitaria ibs.GRANADA, Granada, Spain; ^6^ Department of Human Anatomy and Embryology, Faculty of Medicine, University of Granada, Granada, Spain; ^7^ Excellence Research Unit “Modeling Nature” (MNat), University of Granada, Granada, Spain

**Keywords:** cardiovascular diseases, coronary artery disease, stents, atherosclerosis, cancer, nanotechnology, vascular tissue engineering

## Abstract

Cardiovascular diseases (CVD) are the leading causes of death and disability in the world. Among all CVD, the most common is coronary artery disease (CAD). CAD results from the complications promoted by atherosclerosis, which is characterized by the accumulation of atherosclerotic plaques that limit and block the blood flow of the arteries involved in heart oxygenation. Atherosclerotic disease is usually treated by stents implantation and angioplasty, but these surgical interventions also favour thrombosis and restenosis which often lead to device failure. Hence, efficient and long-lasting therapeutic options that are easily accessible to patients are in high demand. Advanced technologies including nanotechnology or vascular tissue engineering may provide promising solutions for CVD. Moreover, advances in the understanding of the biological processes underlying atherosclerosis can lead to a significant improvement in the management of CVD and even to the development of novel efficient drugs. To note, over the last years, the observation that inflammation leads to atherosclerosis has gained interest providing a link between atheroma formation and oncogenesis. Here, we have focused on the description of the available therapy for atherosclerosis, including surgical treatment and experimental treatment, the mechanisms of atheroma formation, and possible novel therapeutic candidates such as the use of anti-inflammatory treatments to reduce CVD.

## 1 Introduction

Cardiovascular diseases (CVD) are the leading causes of death and disability in the world, with coronary artery disease (CAD) being the most common (Roth et al., 2017). Current high-income countries’ lifestyles, like low physical activity and diets with high fat and glucose content (Kubota et al., 2017; Tappia and Blewett, 2020), contribute to the formation of an atherosclerotic plaque in low diameter vessels such as coronary arteries that supply blood to the myocardium (Libby, 2021a). Atherosclerosis progressively occludes blood vessels and, in certain situations, the atheroma could break after the complete occlusion and form a clot that collapses the vessel (Herrington et al., 2016). In both cases, the patient will suffer a myocardial infarction (MI) that can cause death as a result of the defective irrigation of cardiac muscle by the affected vessels if it is not quickly and properly treated (Tibaut et al., 2017).

Current treatments for atherosclerosis consist of repairing the vascular lumen of the affected vessels to allow a normal blood flow to the compromised muscle (Crea and Libby, 2017). Complementary pharmacological treatments may contribute to a decrease in atherosclerosis progression, for example, by controlling the cholesterol and glucose blood levels as well as trying to prevent thrombosis (Martín-Timón et al., 2014). If the pathology is sustained during a long period without treatment or if the patient suffers MI, the cardiac cell death and adverse cardiac remodelling can lead to terminal heart failure (THF) (Hsich, 2016; Sekuli et al., 2019). The recovery of normal blood flow can be performed percutaneously, by angioplasty, or surgically, by coronary artery bypass (CAB) (Gaba et al., 2021) Figure 1. In the first case, the procedure is minimally invasive, a coronary catheter with a balloon is introduced until the stenotic area, the balloon is inflated and the artery recovers its normal lumen after which the balloon is deflated and removed (Keulards et al., 2020). In most cases, a stent is implanted when the balloon is inflated to keep the vessel open. In the second case, the coronary bypass implies a surgery that uses other healthy autologous vessels, whose replacement is innocuous (usually mammary and saphenous veins or the radial artery (Sánchez et al., 2018), to connect the aorta to a point of the coronary artery distal to the stenotic area. Therefore, this surgical intervention may allow the ‘‘by-passing’’ of the atheroma and the recovery of normal irrigation in the compromised myocardium (Melly et al., 2018). While MI requires a quick recovery of the blood flow to reduce necrosis at maximum, angina represents a non-critical situation which can be solved with slower procedures implying surgical interventions. Thus, angioplasty can be used for both angina and MI whereas CAB is usually reserved for angina (Melly et al., 2018). Even so, these procedures still have important drawbacks to consider. On one hand, stent implantation stimulates vascular wall cells proliferation which promotes re-occlusion of the vessel through a process known as restenosis (Marx et al., 2011). To overcome restenosis and to provide a long-lasting solution, new stent designs have been developed which include drug-eluting stents or bioresorbable stents. On the other hand, although CAB surgery has been demonstrated to be more efficient than angioplasty (Xie et al., 2021), it is still an expensive and invasive procedure and in 5%–40% of cases, the graft can collapse from thrombosis-associated occlusion (MacRae et al., 2016). Moreover, healthy autologous vessels are not always available and there are additional difficulties such as the absence of commercially available small-diameter (<6 mm) vascular conduits needed for coronary artery replacement (Carrabba and Madeddu, 2018).

**FIGURE 1:**
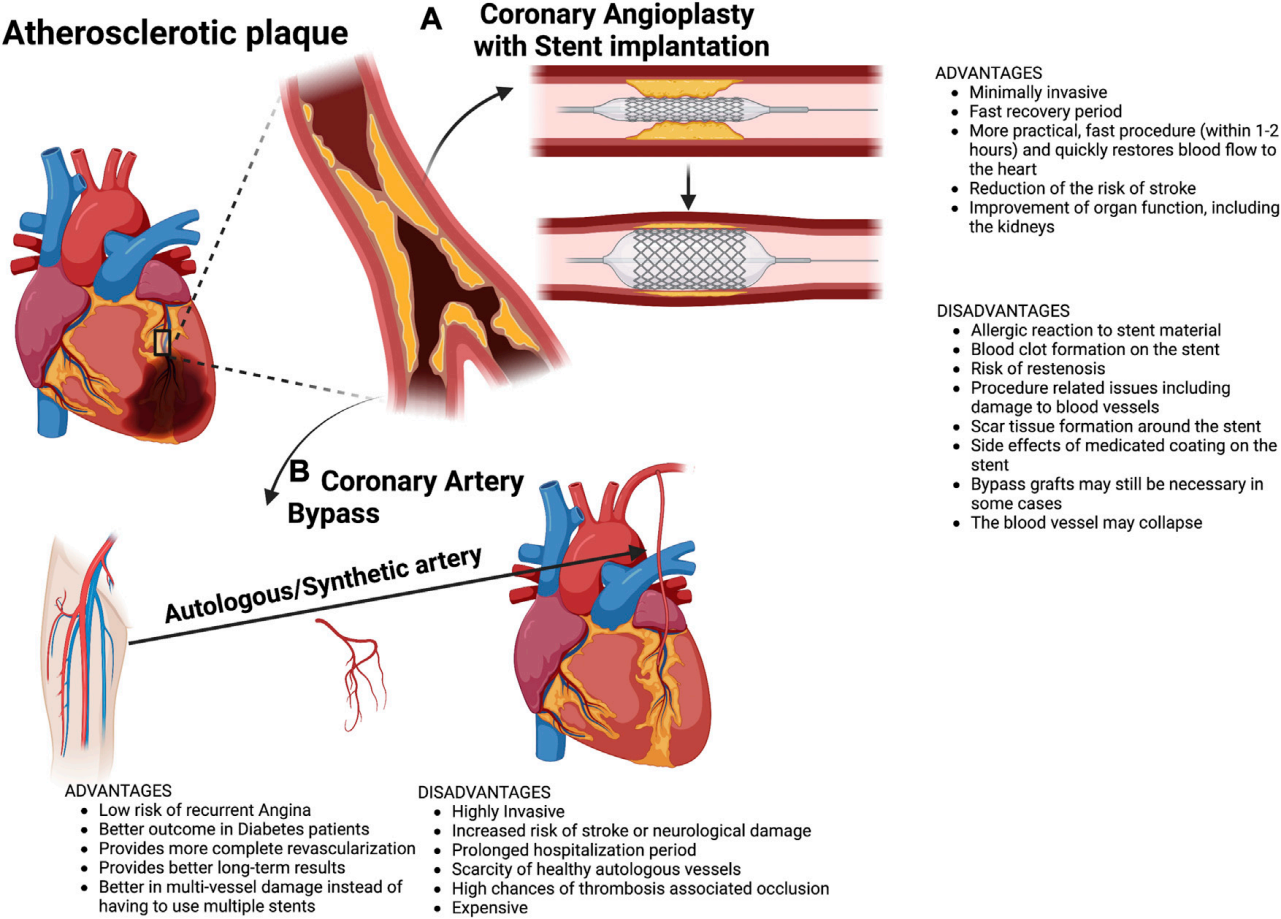
Common clinical procedures to treat coronary occlusion by atherosclerosis. **(A)** Coronary angioplasty with stent implantation and **(B)** Coronary artery bypass, either with an autologous artery or synthetic artery. Main advantages and disadvantages of both techniques are listed.

Comparison of clinical outcomes between percutaneous coronary revascularization vs. coronary artery bypass grafting has revealed that both procedures resulted in similar rates of mortality, myocardial infarction, or stroke (Palmerini et al., 2017).

Because of the current limitations in CVD treatments, revascularization therapies have been focused on biological approaches with the aim of restoring, improving and maintaining tissue function over prolonged periods of time (Karacsonyi and Brilakis, 2017). Some of these novel strategies include the use of nanotherapy to prevent in-stent restenosis, or advances in vascular tissue engineering to develop tissue-engineered vascular grafts (TEVGs). A variety of approaches such as electrospinning and 3D printing have been employed to fabricate TEVGs while others strategies are based on the use of allogenic or xenogenic decellularized scaffolds (Naegeli et al., 2022). Parallelly, developing new therapeutic drugs with the potential to promote the atherosclerotic plaque reabsorption or, at least, mitigate its progression to gain time before obtaining an appropriate TEVG may represent a complementary approach. In this line, it is of key importance to understand the biological nature of the atherosclerotic plaque formation (Manubolu and Budoff, 2022).

Current evidence supports the role of inflammation in the initiation and evolution of plaque, with promotion of cell migration and proliferation that lead to lesion progression (Libby, 2021a). In this respect, atheroma or plaque formation has been described as a kind of neoplasm of vascular smooth muscle origin, establishing similarities between atherosclerosis and cancer (Tapia-Vieyra et al., 2017). Thus, stent implantation represents an external agent that could cause restenosis, but other intrinsic agents such as patients’ genetic alterations could increase the probabilities of developing the atheroma (Dai. et al., 2016; Shlofmitz et al., 2019).

Here we review common CVD treatments and novel therapeutic approaches focusing on the use of nanotherapy and tissue-engineering strategies. Furthermore, atherosclerosis progression and molecular/physiological events that support the correlation between inflammation and atherosclerosis are highlighted in order to open new avenues and opportunities to develop efficient drugs which may prevent and treat atherosclerosis.

## 2 Coronary angioplasty and stent implantation

Coronary angioplasty, developed by Dr.Gruntzig in 1977, was the first method described to perfuse stenotic vessels by recovering the vascular lumen through the inflation of a catheter-guided balloon to the atheroma (Sajadian et al., 2018). The procedure is minimally invasive, relatively non-expensive, effective, and the patient, in most cases, can leave the hospital the same day (King and.ii, 2021). Nevertheless, it has been shown that after a while, angioplasty vessels progressively return to their occluded status in between 30%–40% of the cases (Denktas, 2010) thus, to avoid the abrupt vessel collapse after angioplasty, a little expandable coil or stent is implanted (Sigwart et al., 1987).

**FIGURE 2:**
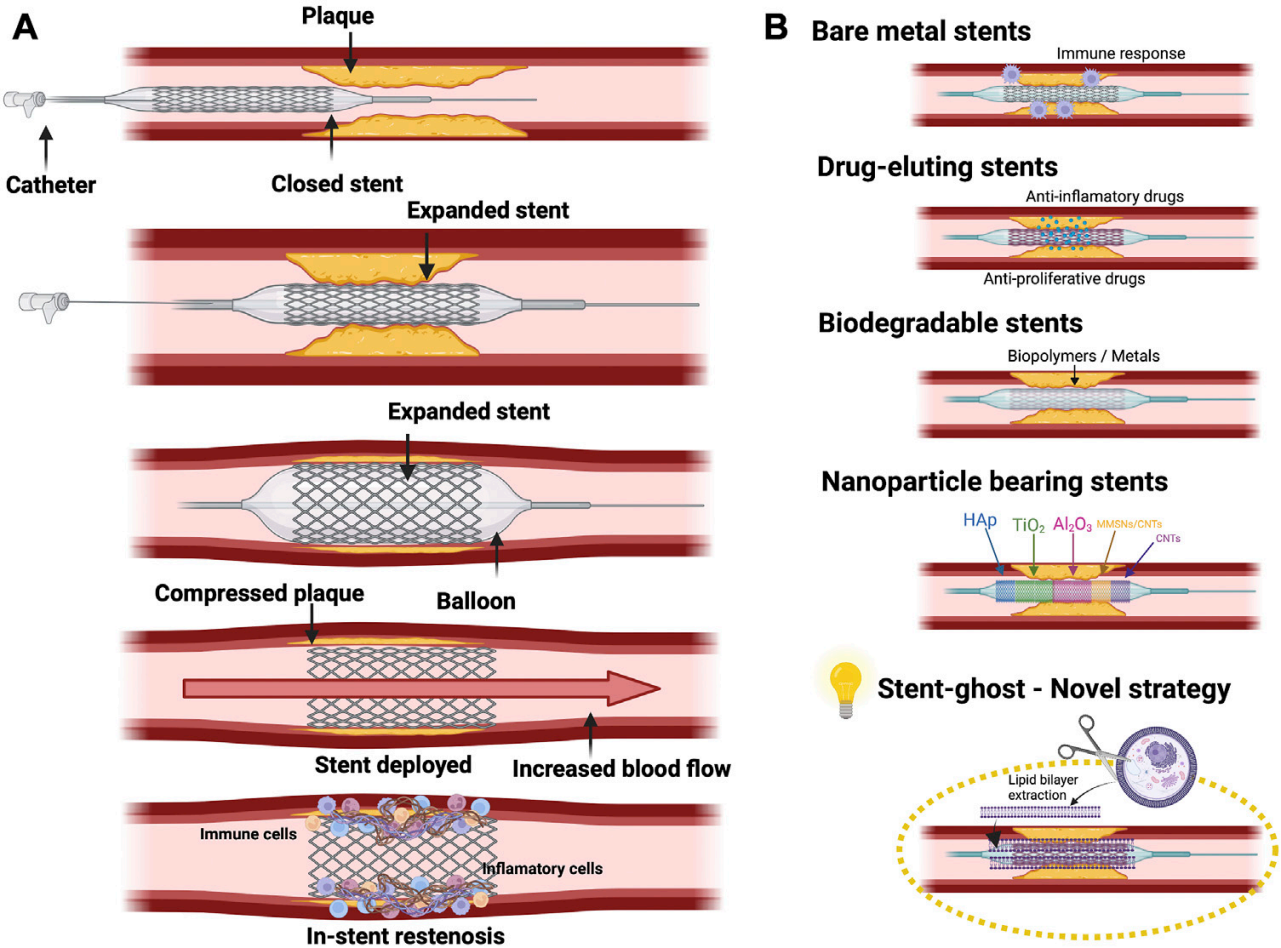
**(A)** Representative stent implantation procedure, including implantation, expansion and lastly, in stent-restenosis formation. **(B)** Representation of different Stents used in clinic: Bare metal stents; Drug-eluting stents; Biodegradable stents; nanoparticle bearing stents. The use of a “Stent ghost” which is a stent coated with cell membranes could represent an innovative therapy to escape the immune surveillance.

It was in 1986 when the first human coronary artery implant was carried out using WALLSTENT® (Schneider AG). This was a structure made of a stainless steel wire-mesh that was self-expanding (Iqbal et al., 2013). Non-etheless, it was taken off the market a few years later due to existing limitations in the stent delivery system which limited its clinical utility. Over the years, many more stents were developed like Multi-link® (Advanced Cardiovascular Systems), Micro® (Applied Vascular Engineering) and Wiktor® (Medtronic) to mention but a few. This was a major advance in the field of coronary angioplasty but it did not come without a number of drawbacks as most of the early stents were bulky and hard to manage technically due to their high metallic density which ultimately led to a high rate of sub-acute thrombosis (Iqbal et al., 2013). Moreover, the mechanical stimuli over the cell wall provoked cytokine release that stimulates cell proliferation and migration to the injury. This phenomenon is named in-stent restenosis and may provoke coronary artery re-occlusion through an inflammatory and proliferative response against the foreign body (Buccheri et al., 2016; Cornelissen and Vogt, 2019). Hence, despite there being reduced restenosis in comparison to the plain old balloon angioplasty, the occurrence of in-stent restenosis was still high because of the migration and proliferation of cells within the stents (Iqbal et al., 2013). In fact, in-stent restenosis was associated to a high mortality and morbidity rate (Drozd et al., 2010). According to these data, restenosis and the immune response against the stent are two of the main hurdles in this therapeutic approach. Remarkably, both events are characterized by a pro-inflammatory response (Iwata et al., 2021), so it seems reasonable to assume that the immune reaction against the stent may be deeply involved in the promotion of in-stent restenosis. With the aim to solve in-stent restenosis associated complications, a new generation of stents including drug-eluting stents (DES) and bioresorbable stents (BS) have been developed (Buccheri et al., 2016). Figure 2 schematically shows the implantation of the stent and the subsequent appearance of restenosis together with different type of stents.

Regarding DES, different compounds have been used to cover bare metal stents (BMS) to target the proliferation of vascular smooth muscle cells (SMC), platelet activation, inflammation and thrombosis. Examples of DES are heparin-coated BMS, used to prevent thrombosis, and BMS loaded with phosphorylcholine which imitates the cell membrane, but the benefits have been limited (Iqbal et al., 2013). DES can also be coated with bioactive compounds that avoid vascular SMC activation and proliferation**,** reducing restenosis development and also modifying the healing process after implantation (Bønaa et al., 2016; Brugaletta et al., 2021). Everolimus, sirolimus and paclitaxel have been shown as antineoplastic agents with a high potential to reduce neointimal overgrowth, maintaining a bigger vascular lumen. Their mechanism of action is based on mTOR inhibition, decreasing cellular anabolic metabolism, growth, division and migration; fundamental pillars of restenosis. Recent studies have shown that there is a clear benefit when using everolimus eluting stents as compared to other BMS in regards to targeting lesion revascularization and the acquired results depending on the patient type and device used (Brener et al., 2013; Sakurai et al., 2013). Apart from the mTOR pathway inhibition, other strategies have been considered to avoid restenosis. For instance, potassium channels blockade, which are necessary for SMC activation (Lasch et al., 2020). In fact, it has been shown that the inhibition of voltage-gated K + 1.3 channels could reduce restenosis by targeting vascular SMC (Bobi et al., 2020; Ye et al., 2020).

Anti-inflammatory drugs are used to inhibit the inflammatory response derived from stent implantation, reducing one of the main events that promote neointimal overgrowth. The promising use of Dexamethasone has been proved in preclinical models and in clinical studies (Radke et al., 2004; Braga et al., 2019). For instance in porcine damaged coronary arteries to study drug delivery to the tissue, with effectivity being seen in the first 28 days (Lincoff et al., 1997), and in canine femoral arteries that showed a significant decrease in the neointimal hyperplasia (Strecker et al., 1998). In other clinical studies, the dexamethasone-eluting stents have shown benefits compared to BMS, demonstrating a significant reduction of major adverse cardiac events at 12 months and in the restenosis/neointimal proliferation rates at 6 months (Lincoff et al., 1997; Strecker et al., 1998; Pesarini et al., 2009; Park et al., 2013). It is important to note that 10% of the patients treated with DES continue to suffer from restenosis and angina. Further, a delay in the healing of the injured vessel and very late thrombosis after DES implantation have been described (van Beusekom et al., 2007; Taniwaki et al., 2016). Therefore, there is still a necessity to develop less harmful stents/drug association to reduce their adverse effects (Wang et al., 2019).

Dual drug-eluting stents (Dual-DES) combine the beneficial effects of various compounds, while reducing individual dosage and associated adverse effects (Zhang et al., 2017; Cha et al., 2020). For instance, the combined effect of sirolimus (antiproliferative) and triflusal (antithrombotic) eluting stents, have been proven, *in vitro* and *in vivo,* to efficiently deliver both drugs, each in its appropriate dose, and to gain greater reduction of restenosis compared to the single drug-eluting stents (Huang et al., 2010). Other example are prednisolone plus sirolimus Dual-DES, in which, prednisolone enhances the effect of sirolimus, achieving a greater reduction of SMC proliferation, restenosis, fibrin expression and inflammation as well as an enhanced re-endothelialization (Lee et al., 2016). Furthermore, Byrne et al. (2009) have proved that Dual-DES (rapamycin and probucol) in patients with CAD showed a higher reduction of restenosis than simple stents.

Bioabsorbable/biodegradable stents (BS) are another new generation of stents that try to reduce injury by naturally dissolving or being absorbed by the body (Erne et al., 2006). These types of stents can be composed of metals, such as magnesium (Mg) or zinc (Zn), or biodegradable polymers such as poli-D,L-lactic acid (PDLLA), poli-L-glycolic acid (PLGA) or poly(L-lactide–co-ε-caprolactone) (PLCL) that can be reabsorbed in the body after some time (Jinnouchi et al., 2019; Beshchasna et al., 2020). BS present an advantage over other types of stents because they eradicate the factor of a foreign material remaining in the body permanently which is essential in avoiding immune reactions and having to remove the stent later on if necessary (Borhani et al., 2018). Obviously, BS have to be made by biocompatible and non-toxic materials (Joner et al., 2018; Zhang et al., 2021a). In this respect, Mg and its alloys have been used in the development of the first metallic BS because Mg is highly biocompatible and also presents low thrombogenicity. In addition, in an aggressive chloride environment like the human body, its degradation is fast (Gu et al., 2009). Other metal alloys containing iron (Fe) or Zn have also been among the pioneer metals used to manufacture BS (McKavanagh et al., 2018; Bowen et al., 2019). In fact, Bowen et al. (2013) described that Zn and its alloys had a slower degradation rate in comparison to Mg and Fe. Combining this with its good biocompatibility and mechanical properties, Zn and its alloys have been shown to be a safer choice to avoid issues associated with Mg and Fe BS since Mg has a faster corrosion rate than Zn and the corrosion of Fe produces non-bioresorbable iron oxides.

The second generation of BS was composed of biodegradable polymers that generate more innocuous products during their degradation than metallic oxides. One of the most frequently used biodegradable polymers is PLLA due to its high biocompatibility (Xiao et al., 2015). In a period of 12–18 months, PLLA is metabolized into Carbon dioxide and water *via* the Krebs cycle with no toxic products resulting from the degradation. The first globally reported fully degradable stent was the PLLA-based BS by Tamai et al., 2000. Down the road, there have been more advances in bioabsorbable polymeric stents in terms of their capability in drug deliverance and the way they are manufactured. Other polymers like PLGA, polyhydroxycarboxylic acids (PHCA), poly(3-hydroxybutyrate) (P3HB) (Williams et al., 2013) and PLCL are under study to assess their biocompatibility and functionality. Further examples of PLLA stents are Tissue Gen, ARTDIVA and Elixir, whose mechanical characteristics have been extensively studied (Charpentier et al., 2015; Zhao et al., 2016; Wang et al., 2022). In fact, it was shown in the study about everolimus eluting PLLA stent Absorb-BVS-System that the mechanical strength of the stent rapidly deteriorated after the first 3 months following implantation (Bil and Gil, 2016). There have also been other polymers like poly(vinylidene fluoride)-hexafluoropropylene (PVDF-HFP) that have been used with second or third generation DES (Waksman, 2017; McKavanagh et al., 2018). Sirolimus and salicylic acid have also been combined with a poly-anyhydride ester to create a BS that has been found to have both anti-inflammatory and anti-proliferative characteristics (Charpentier et al., 2015). Although, there is a number of polymers being used for medical purposes with properties that make them suitable for stent manufacturing, there are still some important issues to resolve, for example, the generation of toxic products, poor mechanical properties and an unsatisfactory degradation rate (McKavanagh et al., 2018). Taking all this into account, BS still do not present enough radial strength and stiffness in comparison with BMS and this can lead to fatigue and fracturing issues after the stent is implanted. In regards to this, research has been carried out to improve the mechanical properties of BS and it has been shown that plasticizing is an effective solution (75). The sterilization techniques used on the stent must also be considered to avoid affecting the molecular weight and crystallinity of the material (Weir et al., 2004).

Bioresorbable drug eluting vascular scaffolds (BVS) are made up of polymers that will disappear after drug elution (Weir et al., 2004). Interestingly, several randomized trials have shown promising results when comparing BVS with traditional DES (Ellis et al., 2015; Nogic et al., 2018). Although the advantage of BVS is the transitivity of the scaffold, while presenting a longer period of time drug delivery, their efficacy and safety over time have not yet been proven due to limited data.

Nevertheless, it is important to remark that bioabsorbable stents have not represented a significant improvement clinically despite their initial promising results. To date, only two bioabsorbable stents are commercially available, Magnesium Magmaris (Biotronik) and polylactic Absorb (Abott) (Mridha et al., 2019). In addition, a recent randomized meta-analysis study comparing the mid- and long-term clinical outcomes of both durable polymer drug-eluting stents (DP-DES) and bioabsorbable polymer drug-eluting stents (BP-DES) revealed no statistically significant differences in cardiac mortality, stent thrombosis, target lesion revascularization, target vessel failure or reinfarction rates (Mridha et al., 2019).

In fact, a conducted trial to compare BVS *versus* metallic evorilimus-eluting stents (EES) did not produce conclusive results, since no significant differences were found between patients from either group in terms of cause of death after 1 year (Tamburino et al., 2016).

The first BVS to be put on the market and to be clinically used was the Absorb BVS (Abbott Vascular, Santa Clara, CA, United States) but even it had limitations in regards to scaffold recoil and thrombosis (Seo et al., 2020). A longer randomized study that tested the differences between BVS and DES was carried out in over 500 patients and there was a noted decrease in angina recurrence and deterioration in patients after 12 months (Caiazzo et al., 2015). None the less, after 3 years, there was in-stent loss and nitrate induced vasomotion observed. The original Absorb BVS had a strut thickness of 150 μm and this has been attributed to being the cause of the adverse reactions observed (Caiazzo et al., 2015). Thus, this led to the development of second and third generation BVS to tackle the issues of the stent’s strut thickness. Therefore, a next-generation of scaffolds with smaller strut thickness were developed, for example, Biolute BRS with a strut thickness of 108 μm and MeRes and DESsolve, both with a strut thickness of 100 μm (Seo et al., 2020). The expected advantages from this new generation of BVS would be lessening the flow disturbances and in the long run, reducing platelet activation and thus the scaffold’s thrombogenicity. Besides the strut thickness of the BVS, another promising research area is reducing the reabsorption time of the scaffold. In this regard, it has been demonstrated that DESsolve scaffolds are bio-absorbed and biodegraded within 1–2 years (Seo et al., 2020).

All this does not come without limitations and as mentioned before, this is a field that is promising but one that requires further research. Kozuma et al., further prove this with a 5 years follow up study they carried out to assess the long-term results of using BVS in comparison to DES that showed comparable results in regards to patient and device based outcomes (Serruys et al., 2015).

In short, there is still a need of more studies to prove the benefits of BVS in comparison to DES since studies carried out in the past 10 years showed BVS´s adverse effects and non-significant improvement in terms of patient´s outcomes in comparison to other types of stents (Serruys et al., 2015; Kozuma et al., 2020).

Other strategies to improve stent hemocompatibility and reduce restenosis are based on the implementation of nanotechnology (Zhao et al., 2016; Botelho et al., 2021; Georgakarakos et al., 2021). The use of nanoparticles (NPs) to inhibit restenosis has been reviewed (Karimi et al., 2016). In short, different types of NPs have been tested, such as liposomes, phospholipid-based micelles; polymeric nanoparticles; hydrogel nanospheres and magnetic nanoparticles leading to reduction of inflammation and angiogenesis (Zhao et al., 2016; Hu et al., 2020).

Interestingly, similar to stents, the clearance of systemically inoculated nanoparticles by immune surveillance also represents a major barrier in the context of nanotherapy which has been extensively assessed (Hu et al., 2020). Thus, from a new perspective, the use of therapeutic nanoparticles, for instance directed against tumour cells, could be compared with the implantation of a stent. In both cases, foreign elements are recognized by the immune system and they cause rejection. Therefore, the advances related to the use of nanoparticles in other diseases can be applied to the field of stent engineering. In this regard, several strategies have been designed in order to avoid immune reaction, for example, the classical nanoconstructs surface grafting with polyethylene glycol (PEG) polymers to reduce their recognition by the reticuloendothelial immune system (Senti et al., 2022). Nevertheless, the authors also highlighted the short-term perspectives of this approach due to the generation of anti-PEG antibodies by the host adaptive immune response. Furthermore, the functionalization of nanoplatforms with CD47, a self-recognition molecule, with the aim of avoiding or reducing their clearance by the innate immune system has also been shown (Pham et al., 2021).

On the other hand, significant advances in the last few years have led to the trend of cell membrane-coated nanoformulations with the ability to biomimic and efficiently evade their removal by phagocytosis (Cai et al., 2022). According to these data, it seems reasonable to suggest the translation of nanotherapy-associated advances, regarding to immune evasion, to the development of new generation stents with reduced restenosis induction and improved biocompatibility. For instance, it may be interesting to look into the development of a CD47-homologous peptide-coated stent to increase its biocompatibility (Inamdar et al., 2020) or even cell membrane-coated stents. To this end, several cell membranes could be used, highlighting autologous endothelial cell-derived membranes in order to fabricate a kind of stent which may simulate a continuity of the host endothelial barrier. Obviously, this is a mere theoretical suggestion which should be experimentally assessed. In this respect, the importance of the cell-derived membrane orientation during the coating process should be remarked upon since it has been reported that the right-side-out orientation is the one which provides the appreciated immune-evasive properties (Bu et al., 2021).

## 3 Tissue-engineered vascular grafts

Currently, the leading, long-term therapeutic strategy to treat severe, but not extremely urgent, obstructions in small-diameter vessels (lower than 6 mm), highlighting coronary arteries, consists of performing bypass grafting using autologous vasculature like the saphenous vein, radial arteries or internal thoracic artery (Sánchez et al., 2018). Nevertheless, bypass grafting involving autologous vessels may not be suitable in many cases due to a range of circumstances (i.e., inappropriate graft size, previous diseases, unfavourable operation history or the multiple nature of the vascular occlusion) (Cai et al., 2021; Georgakarakos et al., 2021). Additionally, other inconveniences should be highlighted, such as the vein graft failure due to stenosis (Botelho et al., 2021). In a particular example, the internal mammary artery is commonly used for bypassing the left anterior descending artery in patients with CAD. In this regard, a clinical trial study revealed that the failure of this procedure had a statistical frequency near 10% and it was associated with some risk factors including vessel stenosis or the presence of additional bypass grafts in the diagonal branch (Harskamp et al., 2016). Indeed, the study established a link between the higher incidence of acute clinical events and an increased rate of repeat revascularization (probably derived from the traumatization of the body because of the external manipulation of autologous vessels) (Harskamp et al., 2016).

Large diameter synthetic grafts that are available on the market usually exhibit an acceptable long-term patency rates, however, small diameter synthetic vascular grafts present limited clinical application, in regards to poor mechanical properties and the appearance of infections, induction of intimal hyperplasia or thrombosis (Weekes et al., 2022). In addition, there is no commercially available synthetic vascular graft for small diameter blood vessels. As an alternative, the replacement of the damaged vasculature by the development of tissue-engineered vascular grafts (TEVGs) which can be generally classified into scaffold-based TEVGs or self-assembled scaffold-free TEVGs has been proposed (Chen et al., 2021).

For instance, an alternative to constructed scaffolds is the use of natural scaffolds which already have all the properties that are needed, hence the use of decellularized vessels. The decellularization strategy relies on obtaining an acellular biological scaffold by removing donor tissue-resident cellular populations in order to minimize adverse host immune rejection, while simultaneously preserving the extracellular matrix and tri-dimensional structure. However, in order to have a practical clinical application as a biological scaffold, the decellularized vessels must be previously re-cellularized, preferably, with the patient´s own cells (Lin et al., 2018). As an example, the decellularization of porcine carotid arteries with the aim of obtaining biological vascular scaffolds feasible to be used as vascular grafts has been described (López-Ruiz et al., 2017; Cai et al., 2021; Heng et al., 2021). Nevertheless, small-diameter TEVGs based on allogenic or xenogenic decellularized scaffolds still have several limitations to be addressed before translation to clinical practice. For instance, the induction of host immune response against the vascular graft, inappropriate physical properties of the graft compared with native vessels which usually promotes the generation of aneurysms, graft-related thrombosis or graft-associated infections (Lin et al., 2018). In addition, developing an appropriate decellularization protocol still remains a matter of debate considering it should effectively remove not only host cells but also immunogenic antigens like α-Gal (Jin et al., 2022).

The development of techniques such as electrospinning and 3D printing have facilitated the fabrication of scaffolds that mimic natural vessels’ structures (Weekes et al., 2022). To date, *in vitro* and *in vivo* experiments have shown encouraging outcomes (Fazal et al., 2021; Rickel et al., 2021). Regarding the use of synthetic scaffolds, the development of a thermoplastic polyurethane synthetic vascular graft, using 3D printing technology, with improved mechanical and biocompatibility features compared with commercially available, non-biodegradable polytetrafluoroethylene grafts has been shown. In this study, the vascular graft was implanted into a rat abdominal aorta model using a patch technique. Despite it not being a full graft technique, the synthetic graft was shown to reduce calcification and thrombus formation in comparison to the standard polytetrafluoroethylene graft 30 days post-operation (Sohn et al., 2021). However, biofabrication techniques, including bioprinting, still have some insurmountable limitations. Microfabrication technologies need to better mimic native vascular anatomy to minimize dimensional disparities at the anastomosis site and TEVG (Henry et al., 2017; Fazal et al., 2021). Along with this, further research with large animals is needed, since only a few studies have been performed (Fang et al., 2021). To date, most of the studies have been conducted on rats and rabbits. Moreover, there is a need for longer *in vivo* studies (>12 months) since the patency rate is still considerably lower than in autologous grafts (Horakova et al., 2018).

In fact, self-assembled, scaffold-free vascular grafting is a novel strategy that takes advantage of recent advances in bioreactors and bioprinting technologies. In this line, Itoh and colleagues patented a procedure to create small-diameter, scaffold-free TEVGs by combining 3D bioprinting and bioreactor-based culturing approaches. First, tubular structures composed of human umbilical endothelial cells, human aortic smooth muscle cells and human dermal fibroblasts were generated using a 3D bioprinter. Then, these tubular structures were cultured in a perfusion system (bioreactor) and matured before their implantation in a rat model (Itoh et al., 2015). Another alternative to constructed scaffolds is the use of natural scaffolds which already have all the properties that are needed, hence the use of decellularized vessels. The decellularization strategy relies on obtaining an acellular biological scaffold by removing donor tissue-resident cellular populations in order to minimize adverse host immune rejection, while simultaneously preserving the extracellular matrix and tri-dimensional structure. However, in order to have a practical clinical application as a biological scaffold, the decellularized vessels must be previously re-cellularized, preferably, with the patient´s own cells (Lin et al., 2018). As an example, the decellularization of porcine carotid arteries with the aim of obtaining biological vascular scaffolds feasible to be used as vascular grafts has been described (López-Ruiz et al., 2017; Cai et al., 2021; Heng et al., 2021). Nevertheless, small-diameter TEVGs based on allogenic or xenogenic decellularized scaffolds still have several limitations to be addressed before translation to clinical practice. For instance, the induction of a host immune response against the vascular graft, inappropriate physical properties of the graft compared with native vessels which usually promotes the generation of aneurysms, graft-related thrombosis or graft-associated infections (Lin et al., 2018). In addition, developing an appropriate decellularization protocol still remains a matter of debate considering it should effectively remove not only host cells but also immunogenic antigens like α-Gal (Jin et al., 2022).

The development of a TEVG is still a challenge due to anatomical complexity. Additionally, the mechanical and biological functions of TEVGs need to be improved for TEVGs to make their way to the clinical use (Konig et al., 2010). One of the main causes of vascular graft failure is the lack of a confluent endothelium due to the thrombogenicity of the graft (Joseph et al., 2022; Weekes et al., 2022). Acellular and decellularized TEVGs are commonly affected by thrombosis and thereby early failure (Owens et al., 2015). A variety of studies have focused on the issue of vascular regrowth after TEVG implantation due to its high complexity. To reduce the thrombogenicity of the TEVG, multiple solutions have been explored including the combination of synthetic and natural materials, the incorporation of anticoagulant molecules (e.g., heparin), or the functionalization with several drugs and factors (e.g., VEGF) to promote endothelialization (Rickel et al., 2021). Recently, a new magnetic approach to accelerate cell retention and improve cellular density which consists of the use of a magnetic hydrogel based on bacterial cellulose to target vascular cells has been proposed (Arias et al., 2018).

Nevertheless, the key to long-term thrombosis prevention is the formation of both the ECs and the SMCs layers. Both layers are critical to prevent intimal hyperplasia and for the function of native vessels (Ju et al., 2017). Moreover, the properties of the polymers must be carefully managed to avoid excessive migration and proliferation of cells which can lead to intimal or neointimal hyperplasia and stenosis, which typically reduces the patency of the vessel graft (Leal et al., 2021).

Given the importance of developing cellularized- TEVGs, different cell sources “to dress” the grafts have been proposed. In this respect, autologous cells are the ideal candidate to minimize host immune reactions against the vascular grafts (Chen et al., 2021). However, using autologous cells, such as, endothelial and smooth muscle cells, still present several hurdles like limited harvesting potential and the inability to reconstitute neo-tissues due to poor *ex vivo* differentiation and expansion rates. To overcome these limitations, the use of human skeletal myoblasts has been proposed as an alternative cell source, taking advantage of their high *in vitro* proliferative capacity (Saito et al., 2021). Another approach could rely on the generation of autologous induced pluripotent stem cells from peripheral blood-derived mononuclear cells and inducing their differentiation towards endothelial and smooth muscle cells to develop TEVGs. This approach can´t be translated clinically but it could be useful as a model of developing pathologic vessels with patient-specific cells to test novel therapeutic agents (Generali et al., 2019).

Interestingly, another alternative to generate highly compatible autologous TEVGs consists of the subcutaneous implantation of tubular mandrels in the own host that will receive the future TEVG. This strategy takes advantage of the host immune reaction against the foreign body (tubular mandrel) around which a fibrotic capsule will be generated. After the maturation of the construct, the formed fibrotic conduit is extracted and used as a TEVG and this is very advantageous because of the short process duration (approximately 4 weeks) and the mechanical strength expressed by the resulting matrix *in vivo* (Qiu et al., 2021). Nevertheless, the clinical translation potential of fibrotic capsule-based TEVGs may be deterred as it represents an invasive procedure highly dependent on either the host physiological characteristics (gender, age or pre-existing diseases like diabetes), the implant location or the implant features (i.e., chemical composition or topography). Additionally, it also requires an extensive period of time for its incubation (several weeks) (Geelhoed et al., 2017), so this therapeutic strategy remains mainly in the pre-clinical field. In fact, efforts are being made to shorten TEVGs production time to under 2 weeks in order to make them suitable for translation to clinical practice (Von Bornstädt et al., 2018). Considering the high recurrence frequency of atherosclerosis, one strategy to reduce the time-limiting factor of TEVGs in the case of disease-recurrent patients could be their initiation at the time of the first therapeutic intervention, immediately after the recurrence of the vascular obstruction, as a preventive measure. Moreover, a second surgical intervention may increase post-operatory complications and significantly reduce patient quality of life (Bianco et al., 2021). Considering the period between the application of the by-pass and noticeable reactions to it in the patient as a crucial clinical determinant, it seems reasonable to remark upon the importance of understanding the biological nature of atherosclerotic plaque formation. Such knowledge may provide us with relevant cues to develop specific drugs with the potential to promote atherosclerotic plaque reabsorption or, at least, mitigate its progression in order to gain time before obtaining an appropriate TEVG.

## 4 Inflammation leads to atheroma formation and tumour cell mass initiation

**FIGURE 3:**
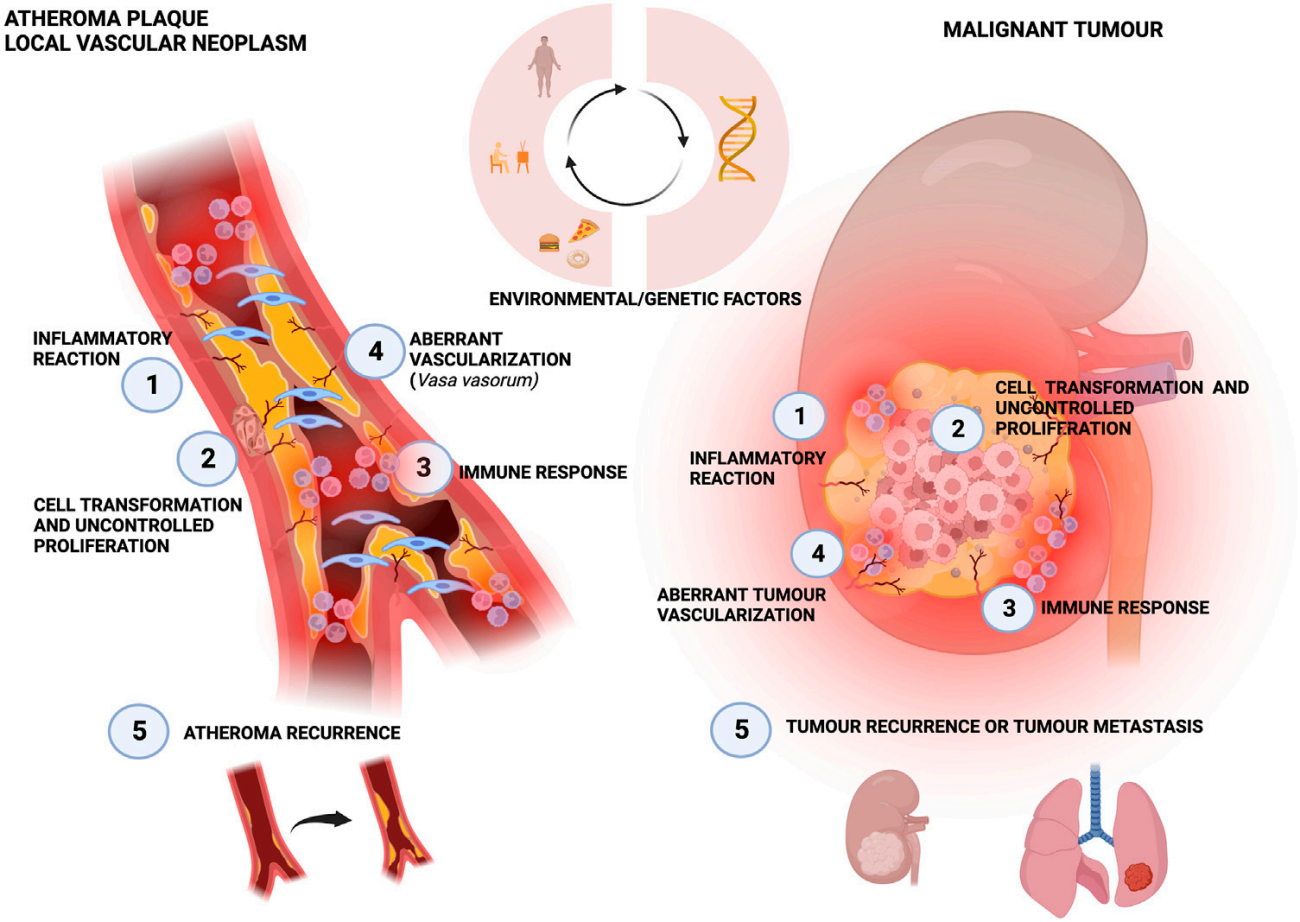
Similarities between atherosclerotic plaque and local neoplasia. The three circular arrows show that both diseases can be influenced by these same risk factors. Both processes are initiated by an inflammatory reaction (Roth et al., 2017), that leads to cell transformation and uncontrolled proliferation (Kubota et al., 2017), which in turn triggers the immune response (Tappia and Blewett, 2020) and an aberrant vascularization (Libby, 2021a). Finally, recurrence is frequently present for both atheroma and cancer (Herrington et al., 2016).

Traditional concepts in which a low-density lipoprotein is the sole cause of atherosclerosis are lately being questioned. The idea that atherosclerosis is a chronic inflammatory disease, with inflammation playing a central role in each stage of the atherosclerotic plaque life cycle has been gaining interest over the past few years (Libby, 2021b). In fact, the use of inflammatory biomarkers has proven to be able to predict the risk of cardiovascular disease before any signals of the appearance of symptoms (Ridker et al., 2018). Similarly, aberrant tumour cell growth is also supported by a background of chronic inflammation which represents a main risk factor for oncogenesis (Liu et al., 2021; Neufert et al., 2021). In this respect, although atherosclerosis and cancer are two different diseases with distinct clinical management, and cancer is commonly associated with genetic mutations whereas atherosclerosis is rather related to environmental causative factors, there are still some similitudes that are relevant to remark (Figure 3). In fact, an interesting point of view relies on considering atheroma formation as a kind of local malignancy rising from the vascular wall (Fasehee et al., 2019), thus being a “wound that never heals” (Garcia-Sabaté et al., 2020). The start point of atheroma formation involves the activation of endothelial cells from a quiescent-like phenotype towards a proliferative one (da Luz et al., 2018) and interestingly, several stimuli can trigger this event through a pro-inflammatory signalling cascade, including LDL accumulation in the subendothelial space (Guo et al., 2021), blood flow disturbance at arterial curvatures or branch points (Chien et al., 2021).

Thus, the relevance of NFkβ pro-inflammatory axis on a molecular level in the activation of endothelial cells to generate atherosclerotic lesions in response to changes in blood flow hemodynamics has been noted (Singh et al., 2021). Remarkably, a link between single-nucleotide genetic alterations and inflammation can be established by focusing on DNA-based deaminases like APOBEC3s family members or AID which induce the transition from Cytosine to Thymine and are enhanced by pro-inflammatory signals (Petljak et al., 2019; Zong et al., 2019). Considering these facts and the documented association between DNA-based deaminases and carcinogenesis (Nishikori et al., 2021), it may be tempting to suggest the existence of an important role of deaminases in atherosclerosis.

Furthermore, the activated endothelium can undergo a process known as endothelial-to-mesenchymal transition (endMT) by which endothelial cells can acquire mesenchymal-like features partially losing their endothelial markers. In fact, the endMT process confers a remarkable phenotypic plasticity since it has been shown that endothelial cells can become smooth muscle-like cells and fibroblast-like cells during atherosclerosis (Zhao et al., 2021). On a molecular level, the importance of the TGFβ/SMAD signalling pathway along with the Wnt2 axis in the progression of endMT, playing a key role in the increase of endothelial cells migration, invasion and neointimal formation has been reported (Zhang et al., 2021b). Moreover, it may be reasonable to propose that the activation of TGFβ and the endMT process may be early events in atherosclerosis since non-laminar blood flow and DNA methylation changes have been associated with the promotion of the endMT process and Wnt/β-catenin signaling pathway in endothelial cells (Björck et al., 2018). Interestingly, the endMT process can be considered as the homologue of epithelial-to-mesenchymal transition (EMT) by which almost all types of tumours exhibit a more aggressive phenotype.

The uncontrolled proliferation of vascular SMC which migrate from the media to the intima vascular layer and contribute to neointimal hyperplasia and arterial remodelling during atherosclerotic plaque progression (An et al., 2022) could resemble oncogenesis. Specifically, vascular SMC undergo a phenotypic switch from a quiescent and contractile phenotype towards a proliferative and migratory one with enhanced capacity to synthesize extracellular matrix components like collagen. Notably, the key role of miRNAs in such a dedifferentiation process by regulating intimal thickening has been reported, with the miR-146b-3p/PIK3CG axis as a representative example (Zhuang et al., 2021). Of note, the central role of transcription factor KLF4 in this phenotypic change triggered by several stimuli including the exposure of contractile vascular SMC to oxidized LDL has been highlighted (Wang et al., 2021). Parallelly, the phenotypic switching undergone by cancerous cells, from a well-differentiated phenotype towards a cancer stem-like one, which could also be termed as a “dedifferentiation process” has been widely described, with crucial roles during tumour progression of enhancing cellular aggressiveness and metastasis (Sandiford et al., 2021). Supporting the homology between vascular SMC and malignant cells, the clonal expansion of vascular SMC which have the potential to originate the majority of the cell types within atherosclerotic lesions has been confirmed (Wang et al., 2020). Interestingly, this fact may resemble the ability of cancer stem cells to generate all cancer subpopulations within a tumour mass. Indeed, the existence of a kind of “atherosclerotic stem cell” with an SMC lineage has already been suggested (Direnzo et al., 2017) which reflects a homologous potential to cancer stem cells.

In the same line, it has been revealed that vascular SMC phenotypic plasticity is far more complex than described above since SMC could even acquire distinct cellular fates, according to some single-cell analysis-based studies, such as myofibroblast-like (Wirka et al., 2019) or macrophage-like phenotypes (Brandt et al., 2022). More importantly, the recruitment of circulating innate immune cells, including monocytes/macrophages, into the intimal vascular layer in an early stage of atheroma biology has been confirmed. Specifically, the activation of endothelium triggered, for instance, by blood flow disturbances can lead to the expression of adhesion molecules, like ICAM1, in the surface of activated endothelial cells which promotes leucocytes extravasation (Murphy et al., 2018). Therefore, these data may suggest the relevance of both fibroblast-like and macrophage-like cells in atherosclerotic occlusions. Similarly, the generation of a complex tumour microenvironment around solid tumour masses, with cancer-associated fibroblasts (CAFs) and tumour-associated macrophages (TAMs) playing significant roles in the support of cancer progression has been widely observed (Tang et al., 2022). Focusing on plaque-associated macrophages (PAMs), it is relevant to note the distinct polarization they can acquire within the plaque, with pro-inflammatory, M1-like PAMs being correlated with atherosclerosis progression and plaque instability/rupture whereas anti-inflammatory/regenerative, M2-like PAMs are correlated with disease regression, plaque stability and better prognosis (Garcia-Sabaté et al., 2020). An equal bimodal polarization can also be identified regarding to TAMs but, intriguingly, with opposite roles since M1 TAMs have been associated with an anti-cancer behaviour while M2 TAMs have been classified as pro-tumorigenic cells (Hernández-Camarero et al., 2021). In any case, macrophage-like cell specific polarization may be a factor of key importance in both disorders. Remarkably, M1-like polarization in atherosclerotic plaque triggered by vascular SMC secretome has been shown to exhibit a diminished potential to recognize and remove opsonized diseased/unwanted cells, like proliferative vascular SMC (Wang et al., 2020). In other words, pro-atherosclerotic vascular SMC can modify immune cells like PAMs in order to escape immune surveillance, in a similar fashion to cancerous cells.

Next, it may be relevant to highlight that the intima-media thickening mentioned earlier, along with the accumulation of pro-inflammatory factors and chemokines in the adventitia vascular layer, could induce oxygen deficiency which may trigger abnormal vasa vasorum neoangiogenesis within atherosclerotic lesions (Li et al., 2021). On the other hand, solid malignancies have also been characterized by strongly enhanced angiogenesis within the tumour mass. Indeed, it is thought that uncontrolled cellular proliferation and growth lead to local lack of oxygen and nutrients which may trigger the formation of abnormal intra-tumour vasculature characterized, for instance, by deficient pericyte coverage (Xu et al., 2021).

Traditionally, the main strategy to prevent/manage atherosclerosis relies on controlling its risk factors, i.e., blood LDL levels, hypertension or life habits like smoking. Although, atherosclerotic occlusion can be removed by atherectomy, a common recurrence of the disease within 2 years after the surgical intervention in up to 50% of patients has been noted (Bath et al., 2021). Interestingly, this fact may be similar to the well-known recurrence of malignant growth after the surgical removal of tumour mass. Thus, it is key to consider atherosclerotic plaque formation as a continuous process which may progress over time, so any effective therapeutic strategy may be a long-term treatment rather than a punctual procedure (Botts et al., 2021).

Considering the implication of the chronic inflammatory response in atherosclerosis, the administration of anti-inflammatory drugs like Omentin-1 has been proposed (Lin et al., 2021).

Recent clinical trials have shown that targeting inflammation can reduce cardiovascular events. The “Canakinumab Anti-inflammatory Thrombosis Outcomes Study” (CANTOS) which was a randomized double-blind trial, targeting the interleukin-1β innate immunity pathway with the monoclonal antibody canakinumab concluded that the antiinflammatory therapy led to a significantly lower rate of recurrent cardiovascular events than the placebo, independent of lipid-level lowering (Ridker et al., 2017a). Although patients treated with canakinumab showed greater incidence of infections, one positive event was the reported highly significant reduction in incident and fatal lung cancer (Ridker et al., 2017b).

In addition, two trials using the natural anti-inflammatory factor Colchicine at different dosages have been conducted. The “Colchicine Cardiovascular Outcomes Trial” (COLCOT) also showed improvements in reducing recurrent cardiovascular events after the development of acute coronary syndromes, but the incidence of pneumonia increased in the treated group. In the second trial, “Low Dose Colchicine 2” (LoDoCo2), the diminution in Colchicine administration also reported a reduction in recurrent events, similar to COLCOT, supporting the role of inflammatory pathways in the pathogenesis of atherosclerosis (Tardif et al., 2019; Nidorf et al., 2020).

Following the rationale of relating atheroma biology with clinical approaches, it might also be tempting to specifically target activated endothelial cells in order to reverse the endMT process (Zhang et al., 2021b; Huang et al., 2021), or specifically focus on inhibiting the dedifferentiation of vascular SMC by promoting the recovery of their contractile phenotype (Shi et al., 2021). Another potential therapeutic strategy could be the enhancing of efferocytosis, the removal of unwanted/pathogenic plaque-associated cells by own phagocytes, in order to induce plaque regression. In this regard, the neutralization of CD47-expressing M1-like PAMs has been proposed with the aim of improving their sensibility to opsonized vascular SMC within atherosclerotic lesions (Wang et al., 2020). Furthermore, the modulation of PAMs phenotype could also be an interesting immunotherapy-based idea. In fact, enhancing M2 PAM polarization and increasing M2/M1 PAM ratio within atherosclerotic occlusion may improve clinical outcomes and plaque regression (Zhang et al., 2021c).

Finally, it has been noted that the abnormal vasa vasorum neo-angiogenesis within atherosclerotic plaque usually leads to the establishment of weak vessels prone to rupture and induce intra-plaque haemorrhages and instability. Thus, the inhibition of vasa vasorum neo-angiogenesis and/or the promotion of the correct maturation of newly formed intra-plaque vasculature could be an interesting therapeutic approach with the aim of reinforcing plaque stability (Li et al., 2021).

## 5 Conclusion

Novel stents have been developed and they have revolutionized interventional cardiology, however, restenosis and immune responses against the stent are still two of the main hurdles in this therapeutic approach. Although improvements have been made in stent design, such as the use of bioabsorbable materials combined with drug delivery, their capacity to maintain tissue function over prolonged periods of time needs to be improved.

Precise and accurate technological advances like electrospinning and bioprinting have allowed the fabrication of new generations of vascular scaffolds. Furthermore, the use of descellularized vessels or *in situ* vessel creation by subcutaneous implantation of tubular mandrels are novel approaches that are being tested in animal models. In addition, advances in strategies to cover stents with therapeutically designed nanoparticles, can be applied in the development of stent-coating to avoid immune rejection. For instance, the use of a CD47-homologous peptide-coated stent or even cell membrane-coated stents in order to fabricate “stent-ghosts” may open a door for new strategies to increase stent biocompatibility and provide the appreciated immune-evasive properties.

Finally, understanding inflammation as the driving force that pushes plaque formation could help to improve common CVD therapies. In addition, our growing knowledge of the biology of atherosclerosis could help to develop novel strategies based on anti-proliferative therapies, cell reprogramming approaches or immunomodulatory treatments to improve prevention and treatment of vascular related diseases.
